# Langerhans cell histiocytosis: unusual dorsal spine localization in an adult male

**DOI:** 10.1259/bjrcr.20220142

**Published:** 2023-01-17

**Authors:** Giovanni Foti, Chiara Longo, Fabio Lombardo, Enrico Piovan, Francesco Colpani, Alberto Beltramello

**Affiliations:** 1 Department of Radiology, IRCCS Ospedale Sacro Cuore – Don Calabria, Negrar, Italy; 2 Department of Neuroradiology, Ospedale Carlo Poma, Mantova, Italy; 3 Department of Pathology, Ospedale Carlo Poma, Mantova, Italy

## Abstract

This case report describes the clinical, imaging, and pathological features of a case of Langerhans cell histiocytosis affecting a patient suffering from chronic thoracic spine pain. Spinal localizations of Langerhans cell histiocytosis have been rarely described and they are usually characterized by involvement of vertebral bodies with osteolytic lesions. Our case presented with several unusual features that delayed the diagnosis, including the age of patient and the involvement of left T10 costovertebral junction with relative sparing of vertebral body and costal bone. The clues for diagnosis were represented by increased signal intensity both on *T*_2_W fat-saturated and *T*_1_W images after administration of gadolinium. The diagnosis was finally confirmed by means of percutaneous biopsy with subsequent histological/immunohistochemical study.

## Introduction

Langerhans cell histiocytosis (LCH) is a rare proliferative disease characterized by infiltration of a single or multiple organs by dendritic cells that resemble the normal epidermal Langerhans cells.^1^ LCH includes a broad spectrum of pathological conditions ranging from the solitary well-treatable eosinophilic granuloma to the disseminated life-threatening variety known as Letterer-Siwe disease.^2–9^ Although LCH is considered a pediatric disease, several cases have been reported in the adult population.^10^ Moreover, spinal localization is usually considered very rare in adults.^10,11^ In the spine, LCH mainly involves the vertebral bodies,^3–6,10^ with a pre-dilection for the thoracic spine (54%) followed by the lumbar (35%) and cervical spine (11%).

We present a case of spinal LCH characterized by several unusual features, as typical osteolysis of vertebral bodies and fludeoxyglucose (FDG) uptake on positron emission tomography-CT (PET-CT) were not present.^12^

## Case report

This case deals about a 50-year-old caucasian malec omplaining for a dull long-lasting pain located in the dorsolateral region of the chest on the left side.

Medical history was unremarkable for trauma or other bone disease. He had no history of fever, night sweats or weight loss. Physical examination revealed limited movement at the waist with no motor or sensor abnormalities. Neurological examination was normal.

Laboratory tests (blood cell count, serum electrolytes, renal and liver function tests, erythrocyte sedimentation rate (ESR) and C-reactive protein (CRP) were normal. Chest X-rays was considered normal.

**Figure 1.**
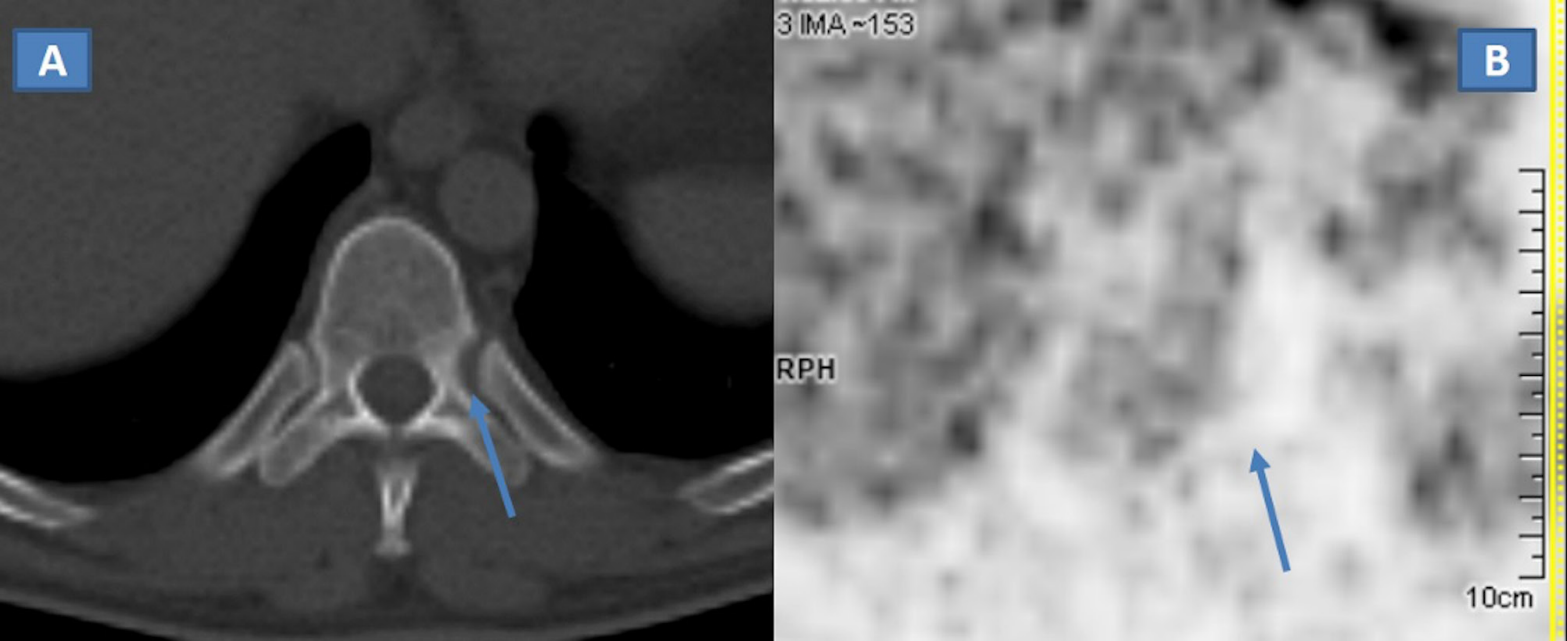
(A, B) Baseline CT scan revealed a widening of left T10 costovertebral junction with scalloping and mild sclerosis of the left aspect of T10 vertebral body (**A**), partially involving the left pedicle and the adjacent costal bone (arrow). The 18-FDG PET/CT on the same level (**B**) was normal, with absence of tracer uptake (arrow). FDG, fludeoxyglucose; PET, positron emission tomography

Baseline CT scan revealed a widening of left T10 costovertebral joint with scalloping and mild sclerosis of the left aspect of T10 vertebral body, partially involving the left pedicle and the adjacent costal bone (Figure 1A). 18-FDG PET/CT showed no significant tracer uptake (Figure 1B).

**Figure 2.**
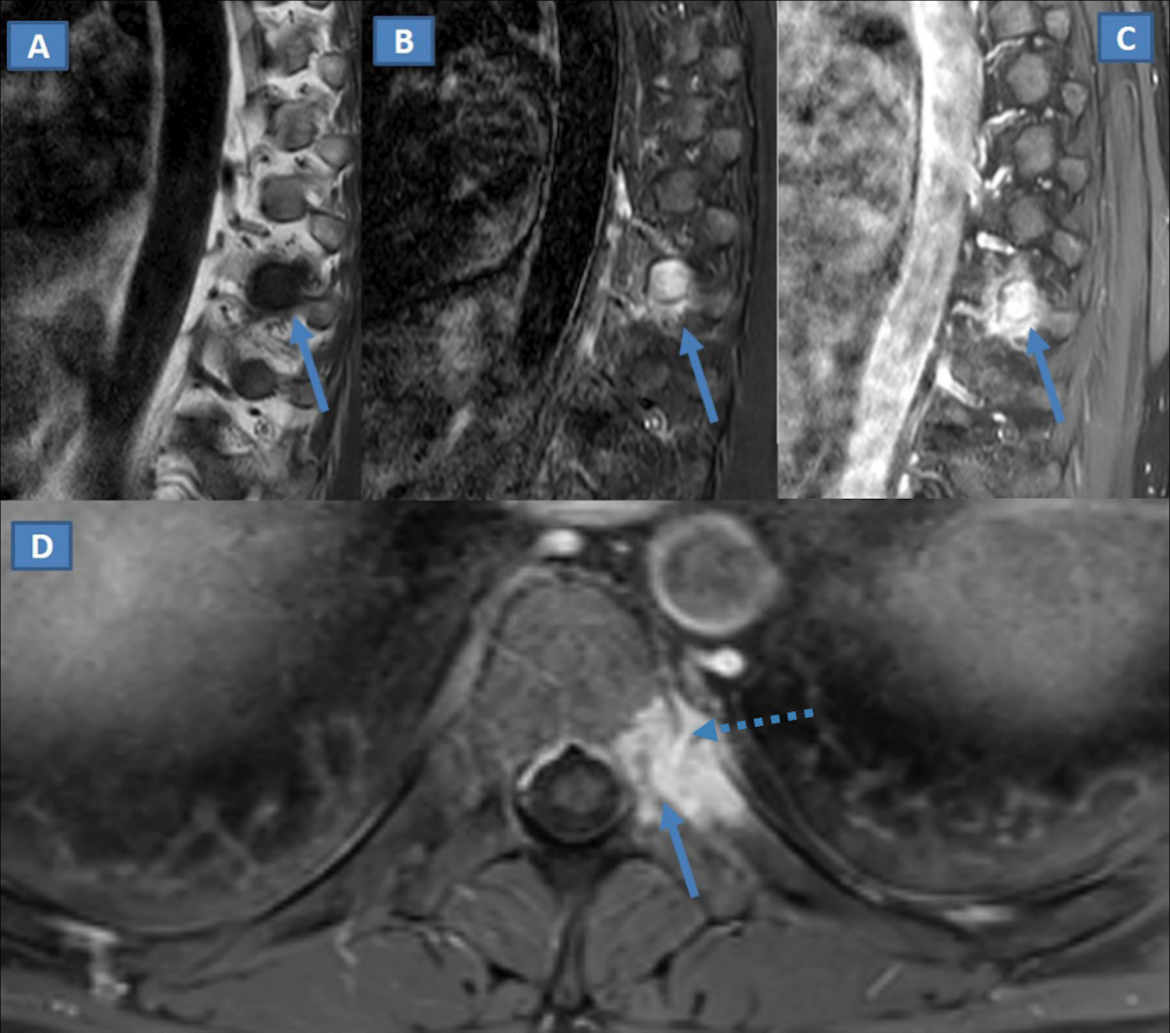
(A–D) On sagittal TSE *T*_1_ weighted MRI at the level of left costovertebral junction (**A**), non-specific signal hypointensity can be depicted (arrow). On the sagittal STIR image on the same level (**B**) signal hyperintensity can be identified (arrow). On the corresponding sagittal (**C**) and axial (**D**) *T*_1_ weighed MR images after intravenous administration of gadolinium, contrast enhancement can be depicted around the T10 costovertebral junction (arrow), with enhancement and subtle widening of articular space (dotted arrow). The enhancement partially involves left costal bone and the adjacent vertebral arch, with relative sparing of vertebral body. STIR, short-tau inversion recovery

MRI revealed non-specific changes of left T10 costovertebral joint, with bone marrow edema of T10 body and pedicle and adjacent costal bone (partially extending to the left neural arch, without spinal canal invasion), characterized by a low signal on *T*_1_W and high signal on *T*_2_W fat-saturated images (Figure 2A and B), with non-specific contrast enhancement depicted after Gadolinium administration (Figure 2C and D).

A fluoroscopy-guided needle biopsy at the level of T10 vertebral body was therefore performed.

**Figure 3.**
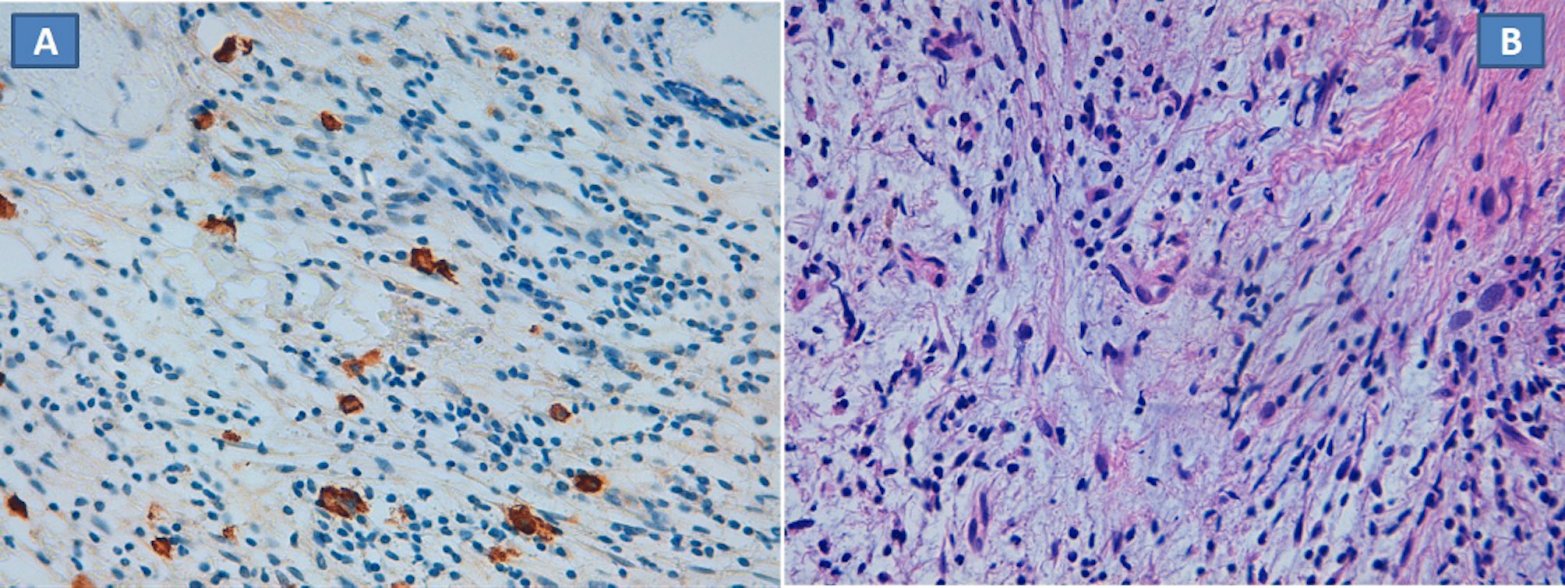
(A, B) Histopathology showed fibrous/ myxoid tissue with reactive lymphocytes and plasma cells associated to rare “histiocytic” elements resembling a non-specific chronic osteomyelitic pattern (A; 400x). A CD1a immunostain (400x) was performed that revealed numerous Langerhans cells diagnostic of LCH (**B**). LCH, Langerhans cell histiocytosis.

Histopathology showed fibrous/myxoid tissue with reactive lymphocytes and plasma cells associated to rare “histiocytic” elements resembling a non-specific chronic osteomyelitic pattern (Figure 3A). On the basis of a pathological suspicion of LCH, a CD1a immunostain was performed that revealed numerous Langerhans cells, diagnostic for LCH (Figure 3B).The absence of eosinophils associated to the non-specific pattern at histopathology was in favor of the possibility of non-active/regressed lesion, considering also the atypical presentation in the adult age.

Patient was referred to the Hematology Department where, considering also the non-active pathological pattern, was successfully treated conservatively.

## Discussion

LCH is a rare condition, usually affecting pediatric population. Spine can be involved in a minority of cases, being the thoracic vertebral bodies the preferred sites, and joint involvement is extremely rare. Usually, in LCH spinal lesions occur in a single vertebral body without extension to neural arch or paravertebral/epidural soft tissues. In advanced stages, the typical complete or incomplete collapse of the vertebral body can be seen, causing anterior wedging with the characteristic “vertebra plana” appearance; this appearance is typical of children, as in adults only a relatively small volume of the vertebral body is affected so that vertebral collapse usually does not occur.^13^ Despite this, in rare cases, posterior arch involvement can be present, as well as paravertebral and/or epidural soft tissue masses.^14^ As a result of the low frequency with which spine lesions are encountered in clinical practice, there are few studies in the literature focusing on this specific disease.

In our case, radiological presentation was unusual, lacking the typical osteolytic pattern. CT showed only a subtle, non-specific widening of left T10 costovertebral junction with mild sclerosis of the adjacent bones that could be easily misdiagnosed in case of lack of chief complain and detailed clinical data. Also, the lesion did not show any FDG uptake at PET-CT. The lack of tracer uptake could be explained because of the absence of significant structural changes and absence of significant inflammation. However, normal PET/CT scan was of great value in excluding the majority of tumoral entities, narrowing the differential diagnosis. MRI changes were non-specific as well, and somehow similar to those of a spondylodiscitis or inflammation of costovertebral joint. However, lab data were normal, without any signs of inflammation. The prevalent involvement of pedicle and costovertebral joint, with relative sparing of vertebral body, is another feature that needs to be underlined.

In our case, there were no signs of osteolysis, both at CT and MRI, and imaging findings resembled those of inflammation or bone marrow edema. Differential diagnosis included other conditions that can involve costovertebral joints as infections (*e.g.* tubercolosis), inflammatory disorder (spondyloarthritis), primary tumors and metastases. In the majority of these cases, however, associated imaging findings are present.

This case demonstrates that LCH may involve any age and any level of the spine. Osteolysis and FDG-uptake on PET-CT of the involved segments are not always present and the radiologist should be aware of atypical or unusual radiographic presentations of the disease when evaluating the spine for evidence of Langerhans, such as involvement of the costovertebral junction. In our case, MRI was of paramount importance because MRI not only revealed the involvement of bone marrow of the vertebral body but also demonstrated the absence of disc involvement, excluding spondylodiscitis. The combination of MRI findings and ancillary changes at CT, including articular space narrowing and subtle sclerosis, could represent a clue for diagnosis.

Final diagnosis relies on pathological findings with the caveat that the lesion can be suspected on the basis of conventional staining, but only immunohistochemistry, revealing the presence of Langerhans cells stained with CD1a - normally never found in normal bone marrow—can establish the right diagnosis. Another remarkable point is that the biopsy should be performed on a relatively spared area of the vertebral body, avoiding the risk of collecting necrotic or inflammatory tissues around the area of costovertebral joint.

A variety of treatment modalities for spinal LCH have been reported, including conservative management, intralesional steroid injection, radiation therapy, chemotherapy, currettage and surgery in selected cases with mechanical instability or neurological deficits due to spinal cord compression, keeping in mind that spinal LCH is self-limiting and the prognosis is usually good.^11,14,15^

## Learning points

- LCH is a rare proliferative disease usually affecting pediatric patients, although several cases have been reported in the adult population.
- Spine can be involved in a minority of cases, with thoracic vertebral bodies being the preferred sites.
- Osteolysis and FDG-uptake on PET-CT of the involved segments are not always present, and imaging findings may only include non-specific signs of inflammation.
